
JOURNAL INFORMATION
==============================
NLM Title Abbreviation: ScientificWorldJournal
Journal ID: ScientificWorldJournal
NLM Journal ID: 101131163
Journal ID: TSWJ

The Scientific World Journal

ISSN: 2356-6140
EISSN: 1537-744X
Publisher: Wiley

ARTICLE INFORMATION
==============================
PMCID: PMC6083889
PMID: 30147628
DOI: 10.1100/tsw.2001.467
Article ID: 284280
Article version: 1
Subjects: Commentary

The SETAC Meeting: Emerging New Directions in Environmental Toxicology

Manning William J.
University of Massachusetts, Amherst, MA, USA
Publication date: 2001
Electronic publication date: 2001 Dec 18
Volume: 1
First page: 914
Last page: 915
Copyright: Copyright © 2001 William J. Manning.
Copyright year: 2001
License: This is an open access article distributed under the Creative Commons Attribution License, which permits unrestricted use, distribution, and reproduction in any medium, provided the original work is properly cited.
License URL: https://creativecommons.org/licenses/by/3.0/

Keywords: evolutionary toxicology, plant bioaccumulation, toxicogenomics

==============================
Commentary
*TheScientificWorld* (2001) 1, 914–915
ISSN 1532-2246; DOI 10.1100/tsw.2001.467

### The SETAC Meeting: Emerging New Directions in Environmental Toxicology

William J. Manning
*University of Massachusetts, Amherst, MA*

*Received November 30, 2001; Accepted December 3, 2001; Published December 18, 2001*

**KEY WORDS:** evolutionary toxicology, plant bioaccumulation, toxicogenomics

**DOMAINS:** environmental toxicology

I joined a happy throng of environmental toxicologists in Baltimore, Maryland at the 22nd annual meeting of the Society of Environmental Toxicology and Chemistry (SETAC) from 11–15 November, 2001. It was a well-attended meeting of enthusiastic people that included an encouraging and healthy number of lively junior investigators and graduate students.

Many of the numerous paper and poster sessions focused on traditional areas, such as aquatic toxicology, bioassays, biomarkers, biomonitors, endocrine disruptors, metals, pesticides, persistent organic pollutants, risk assessment, and sediment chemistry and toxicology. Many of the presentations were from investigations conducted under reductionist conditions.

It was also evident from the program that new directions in environmental toxicology were emerging. Change and new things were in the air. This was evidenced by sessions devoted to evolutionary toxicology, molecular/cellular toxicology, phytoremediation, plant bioaccumulation and phytotoxicology, toxicogenomics, and urban impacts. I will feature developments in evolutionary toxicology, phytotoxicology, and toxicogenomics.

In the evolutionary toxicology session, researchers from Virginia reported that they have demonstrated genetic adaptation in *Fundulus heteroclitus* to PAH in a contaminated estuary, using embryo assays for cardiatric abnormalities. Populations from clean and contaminated estuaries were compared and fewer abnormalities occurred in the assays from the PAH-contaminated estuary, indicating adaptation to PAH. Genetic differences between the two populations were determined using allozyme and m-DNA information.

The number of phytotoxicologists who participate in SETAC meetings is increasing. There are more high-quality posters and papers in the area of plant bioaccumulation or phytoremediation, involving terrestrial plants. Some very nice work from Lancaster University, U.K., identified a new method of plant uptake of semi-organic compounds (SOCs). Conventional wisdom said that SOCs were exclusively taken up by leaf cuticles and then slowly diffused into plant tissues. The Lancaster group established an active and important role for stomata in uptake of SOCs during gas exchange. Plants are becoming increasingly popular in environmental toxicology.

In my opinion, the most important and interesting session at the meeting was the one on toxicogenomics. This was new session for SETAC and unfortunately it was held in one of the smaller meeting rooms. I was fortunate to get a seat as it quickly became a standing room only session.

Email: aamanning@mindspring.com
©2001 with author.

Toxicogenomics provides a new end-point for environmental toxicology. By combining some of the methods of molecular biology, such as DNA sequencing and genomic techniques, with those of environmental toxicology, it is possible to compare gene expression in organisms exposed to stressors or toxicants with those of nonexposed controls. This allows identification of genes and gene products up-regulated in stress responses. It may also allow identification of biochemical pathways that are the most susceptible to specific toxicants. Patterns of gene expression could be used to determine which stressors cause specific toxic impacts, and at what concentrations.

In an interesting example, stress gene expression in corals is being characterized by using a series of probes. Use of this genomic information could allow relation of temporal patterns in gene expression in natural coral populations with specific stressors, such as PAHs and mercury.

This is the age of genomics. We know the human genome and all or much about that of organisms as diverse as fruit flies, zebra fish, *Arabidopsis, Caenorhabditis,* and *E. coli.* Genomic analysis being applied to many more organisms. The combination of genomic techniques with environmental toxicology results in powerful new and precise end-points for the response of organisms to toxicants and environmental stressors. Toxicogenomics is in its initial stages of development, but I am sure it will soon become a key element in environmental toxicology.

**This article should be referenced as follows:**

Manning, W.J. (2001) The SETAC meeting: emerging new directions in environmental toxicology. *TheScientificWorld* 1, 914–915.
